# Comparison of the Effectiveness of Fractional Carbon Dioxide Laser and Retinoic Acid Peel in the Treatment of Acanthosis Nigricans: A Randomized Controlled Trial

**DOI:** 10.7759/cureus.86047

**Published:** 2025-06-15

**Authors:** Ramachandran Gnanasuriyan, Srikanth Shanmugam

**Affiliations:** 1 Dermatology, Mahatma Gandhi Medical College and Research Institute, Puducherry, IND

**Keywords:** acanthosis nigricans, anasi score, chemical peel, dermoscopy, fractional carbon dioxide laser, retinoic acid peel

## Abstract

Introduction

Acanthosis nigricans (AN) is a common skin disorder with varied aetiology and a wide palette of treatment options. Despite multiple options available for management, laser as a modality has recently evolved and shown better outcomes. Some fractional ablative lasers induce a very narrow tissue column of thermal damage and neocollagenesis, thereby improving textural irregularities, whereas retinoids, a first-line drug, help in superficial skin peeling. The study was conducted to determine the efficacy of fractional carbon dioxide (CO2) laser and retinoic acid peel and to decide which is a better and safer treatment option to address AN.

Methods

This is a prospective randomised controlled trial. A total of 38 patients were included in this study and divided into two equal groups. Group A was treated with fractional CO2 laser(n=19) and group B was treated with retinoic acid peel (n=19) using the sequentially numbered, opaque, sealed envelope (SNOSE) technique via allocation concealment method and a computer-generated random number table. Group A received four sessions of laser treatment at two-week intervals, and group B received four sessions of peel treatment at three-week intervals. Both groups were analysed using the acanthosis nigricans area and severity index (ANASI), dermoscopy, and two blinded dermatologists before and one month after the treatment. MedCalc software version 19.0.5 (MedCalc Software, Ostend, BEL) was used for statistical analysis.

Results and conclusion

There was a high statistically significant difference noted in the mean ANASI score in both groups. The fall in ANASI score that denoted the treatment response was greater in group A compared to group B, signifying superior results of fractional CO2 laser compared to retinoid acid peel. The Fractional CO2 laser was more efficacious than retinoic acid peel in the management of AN with sustainable results in the long term, minimal side effects, and required fewer sessions.

## Introduction

Acanthosis nigricans (AN) is a skin disorder characterised by the presence of velvety hyperpigmented thickened plaques in the flexures of the body, such as the nape of the neck, axilla, groin, and sometimes the face. The prevalence of this condition varies from 49.2% to 58.2% worldwide and 61.54% in the Indian population [1,2]. It is usually associated with endocrine disorders like diabetes mellitus and insulin resistance, and rarely with malignancy. It can also occur as a side effect of a few medications like oral contraceptive pills and hormonal treatments, systemic corticosteroids, nicotinic acid, etc. Genetic factors also play a role [3]. The primary treatment aims at treating the underlying disorder. However, the lesions of AN don't reverse completely and produce a cosmetic disfigurement to the patient and require an additional treatment, which helps in the resolution of the lesion, thereby improving their quality of life.

A variety of medications are available for this condition, with varied results. Despite the promising results of lasers and chemical peels in AN treatment, there is a lack of comparative studies, particularly in the Indian population, to determine the most effective and safe treatment option. The fractional ablative lasers, like carbon dioxide(CO2) and 1550nm erbium fiber, improve textural irregularities and pigmented lesions by inducing a very narrow tissue column of thermal damage and ablation of the dermal wound and neocollagenesis. Retinoic acid peel causes thinning and compression of the stratum corneum, accelerating the cellular turnover of keratinocytes, reversal of epidermal cell atypia, dispersion of epidermal melanin pigment, neocollagenesis, and new vascular formation.

These treatment modalities were selected for comparison as both induce superficial exfoliation of the skin. However, existing studies have demonstrated the effectiveness of both approaches in treating AN, with fractional CO2 laser often showing superior clinical outcomes [4]. Dermoscopy, a non-invasive tool, can directly visualize the morphological characteristics of the skin lesions and can be used in evaluating the response of AN to treatment [5]. Thus, we conducted this comparative intervention study to compare fractional CO2 laser and retinoic acid peel in AN and assessed the results both clinically and via dermoscopy.

## Materials and methods

This is a prospective randomized controlled trial conducted by the Department of Dermatology, Venereology, and Leprosy of Mahatma Gandhi Medical College and Research Institute (Puducherry, IND). Based on findings from a previous study [4] and considering a 95% confidence level and 80% power, using the two-sample t-test sample size formula, the estimated sample size was 38 participants, with 19 individuals allocated to each group. This study was approved by the Institutional Human Ethics Committee of Mahatma Gandhi Medical College and Research Institute (approval no. MGMCRI/2024/184/04/IHEC/94) and has been registered in Clinical Trials Registry-India (CTRI), Indian Council of Medical Research (ICMR)-National Institute of Medical Statistics (NIMS) (registration no. CTRI/2025/04/084666).

Patient selection

Patients of both sexes aged 18 to 60 years with a clinical diagnosis of AN of the neck were included. Patients with any skin infection or history of herpes simplex infection on the study site, history of applying retinoids, hydroquinone, corticosteroids, depigmenting agents, those taking oral contraceptive pills, nicotinic acid, corticosteroids, estrogen, niacin, protease inhibitors within one month before the study, with a history of taking systemic retinoids within six months before the study, patients with endocrine disorders, keloidal tendency, a history of allergy to tretinoin and topical anaesthetic creams, and pregnant and lactating women were excluded from the study. 

Patient assessment

The procedure and the steps of the study, side effects, and the expected results were clearly explained to the patients before enrolling them in the study. Written informed consent was obtained from all the enrolled patients for both treatment and clinical photographs. A proper history and general and dermatological examination to rule out any underlying disease associated with AN were done. The clinical assessment of AN was made using the acanthosis nigricans area and severity index (ANASI) scoring system (Table 1). Clinical and dermoscopic pictures of the neck were taken using a smartphone camera (12 megapixels). The dermoscope used was DermLite HUD Dermoscopy (polarized light, 10x magnification; MoleMax Systems, Leichhardt, AUS). All the procedures were done solely by the principal investigator. 

**Table 1:** The ANASI scoring system ANASI: Acanthosis nigricans area and severity index

ANASI score calculation				
Area index (A)				
1	2	3	4	5
<10%	10%-29%	30%-49%	50%-69%	70%-100%
Severity index = Pigmentation + thickening				
Pigmentation (P)				
0	1	2	3	4
Absent	Mild	Moderate	Marked	Severe
Thickening (T)				
0	1	2	3	4
Absent	Mild	Moderate	Marked	Severe
ANASI score = A x (P+T)				

ANASI scoring system

Area

The total area of the neck is calculated by multiplying the length (measured from a point at the junction between the chin and upper neck in full neck extension to a point at the interclavicular space) and the width of the neck (circumference of the neck: measured at the point of junction between the chin and upper neck). Then, the affected area is calculated by multiplying its largest length by its largest width. A percentage is then calculated, and a value is chosen as shown in Table 1.

Pigmentation and Thickness

**Figure 1:**
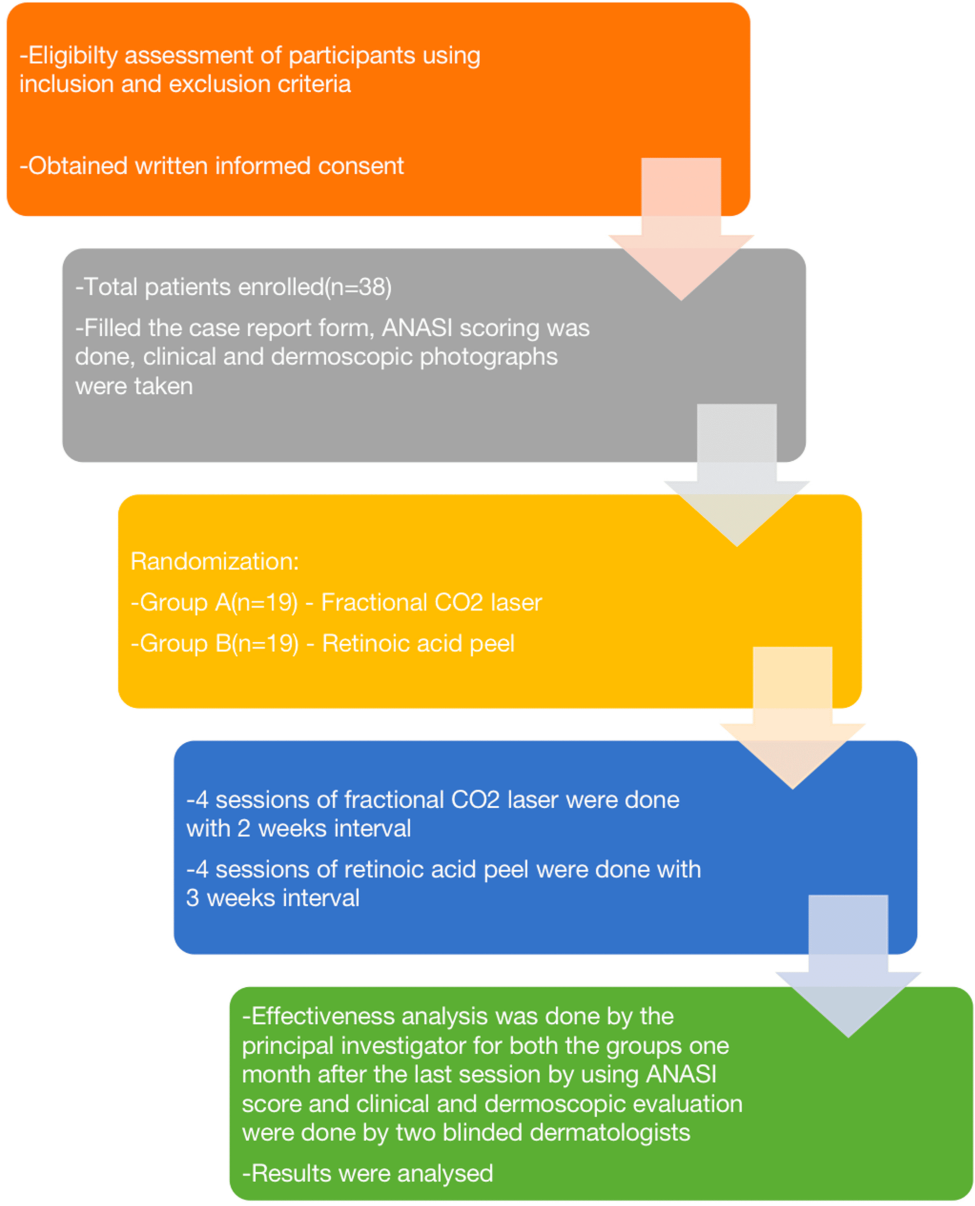
Flowchart summarizing the study procedure ANASI: Acanthosis nigricans area and severity index

Grading is given for pigmentation and thickness (Table 1), and their values are summed up and multiplied by the area value to get the ANASI score. Based on the score, the patients were classified into three subgroups corresponding to the severity of AN as: ANASI score <10 = mild; ANASI score >10 to <20 = moderate; ANASI score >20 = severe. The dermoscopic features of AN were assessed by two blinded dermatologists who compared before and after treatment photographs. The typical dermoscopic signs of AN are sulci cutis, cristae cutis, brown-to-dark brown dots, and milia-like cysts. The patients were split into two groups, namely group A (fractional CO2 laser; n= 19) and group B (retinoic acid peel; n= 19) by sequentially numbered, opaque, sealed envelope (SNOSE ) technique using allocation concealment method and a computer-generated random number table (Figure 1).

Treatment protocol

The fractional CO2 laser was performed in the laser room with necessary precautions. Retinoic acid peel was applied in the minor operation theatre (OT) of the dermatology department. All the patients’ data were documented in a Microsoft Excel sheet (Microsoft Corp., Redmond, WA, USA). Statistical analysis was done using MedCalc software version 19.0.5 (MedCalc Software, Ostend, BEL).

Group A (Fractional CO2 Laser)

The site to be treated was applied with topical anaesthetic cream (containing lidocaine and prilocaine) and occluded. After 45 minutes, the skin was cleansed with saline-soaked cotton pads till it appeared clean from all the dirt and the eyes were covered with wet gauze pads and an eye shield for safety. Superficial peeling and ablation of the affected region was done by fractional CO2 laser using the parameters power 10 W, dwell time 300 μs, spacing 350 µm and two stacking with one pass. After the laser session, an ice pack was applied immediately for cooling. Any immediate side effects, like erythema, erosion, ulceration, and burn, were noted. The patient was advised to apply fluticasone propionate cream and emollient for three days following the procedure. The same procedure was repeated four times at an interval of two weeks.

Group B (Retinoic Acid Peel)

The sites to be treated were degreased with acetone. Following this, retinoic acid 4% chemical peel was applied homogenously by brushing over the affected region till the skin acquired a yellowish colour to visualize the agent. The peel was left for four hours and washed by the patient. The same procedure was repeated four times at an interval of three weeks.

Methods of evaluation 

The ANASI scoring and dermoscopic evaluations were performed solely by the investigator. Clinical and dermoscopic photographs of the lesions were captured before initiating treatment and one month after the final session using a smartphone camera (12 megapixels). Standardized patient positioning was maintained throughout, and patient identity was masked in all images. Clinical photographs were taken from a fixed distance of 30 cm under ambient room lighting without flash. For dermoscopic imaging, a contact dermoscope was placed perpendicularly (at a 90° angle) to the skin without applying pressure. The response to the treatments was assessed by two blinded dermatologists at the end of the study by comparing the clinical and dermoscopic photographs taken before and after the therapy. The results were graded as a percentage of improvement (0% to 100%).

Statistical analysis

MedCalc software version 19.0.5 was used for statistical analysis. Mean, median, standard deviation, and inter-quartile range (IQR) were used for analyzing quantitative data. The Mann-Whitney U test was used for comparing the quantitative data between the independent groups, and the Wilcoxon signed-rank test was used for comparing the quantitative data between the paired groups with a 95% confidence interval and 5% as a margin of error. A p-value of <0.05 was considered to be significant. Fisher’s exact test was used to compare categorical data between two groups.

## Results

Demographic details and disease severity of the study population

A total of 38 patients completed this study, with 19 in each group. Group A (fractional CO2 laser) had nine males and 10 females, with ages ranging from 19 to 55 years, with a mean age of 33.05 ± 10.84 SD years. Group B (retinoic acid peel) had 10 males and nine females with ages ranging from 20 to 53 years, with a mean age of 33.84 ± 10.61 SD years. The total duration of the disease ranged from two to 10 years in group A (median four years, IQR 3-5) and from two to 12 years in group B (median six years, IQR 3- 8). In group A, 52.6% of participants were Fitzpatrick skin type IV and 47.4% were type V, while in group B, 57.8% were type IV and 42.2% were type V. The BMI range was 25 to 43.3 kg/m² in group A (mean: 30.22 ± 4.99 SD) and 23.3 to 44 kg/m² in group B (mean: 30.24 ± 4.77 SD). Notably, two participants in group A and one participant in group B were classified as extremely obese. The severity of AN was assessed using the ANASI scoring system and categorized as mild, moderate, or severe. In group A, two patients had mild AN, 15 had moderate AN, and two had severe AN. In group B, four had mild AN, 14 had moderate AN, and one had severe AN.

Clinical response assessment using the ANASI score

**Figure 2:**
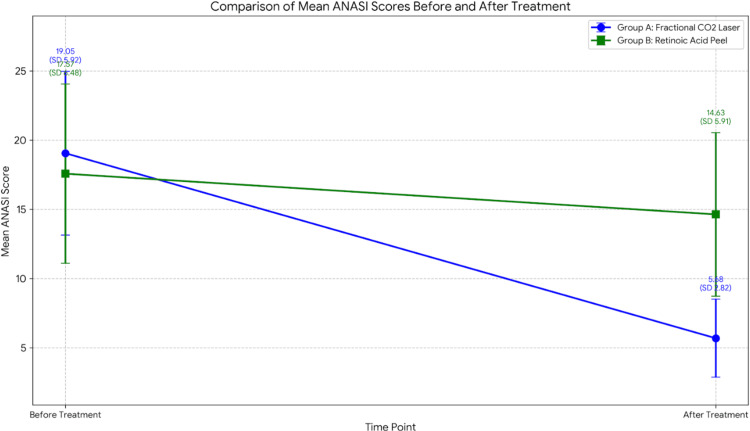
Line graph comparing the mean ANASI score before and after two modalities of treatment ANASI: Acanthosis nigricans area and severity index, CO2: Carbon dioxide

The mean ANASI score before therapy was 19.05 ± 5.92 SD with a median of 18 (IQR 15-20) in group A and 17.57 ± 6.48 SD with a median of 18 (IQR 15-20) in group B. After therapy, this reduced to 5.68 ± 2.82 SD with a median of 4 (IQR 4-8) in group A and 14.63 ± 5.91 SD with a median of 16 (IQR 12-17.5) in group B (Figure 2).

**Figure 3:**
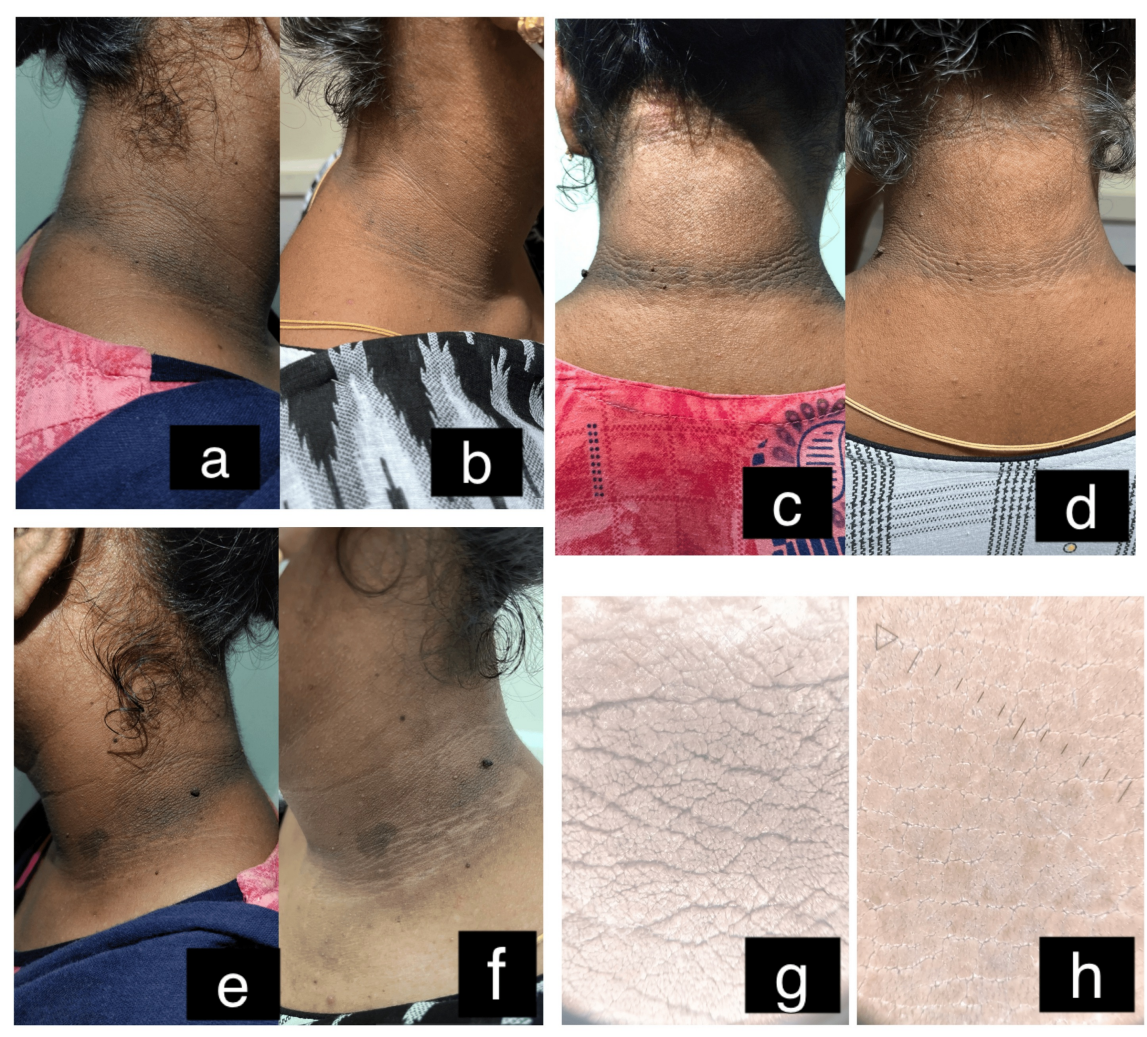
Before and after fractional CO2 laser treatment a: Right side of the neck before treatment; b: Right side of the neck after treatment; c: Nape of the neck before treatment; d: Nape of the neck after treatment; e: Left side of the neck before treatment; f: Left side of the neck after treatment; g: Dermoscopic picture of the neck before treatment; h: Dermoscopic picture of the neck after treatment

**Figure 4:**
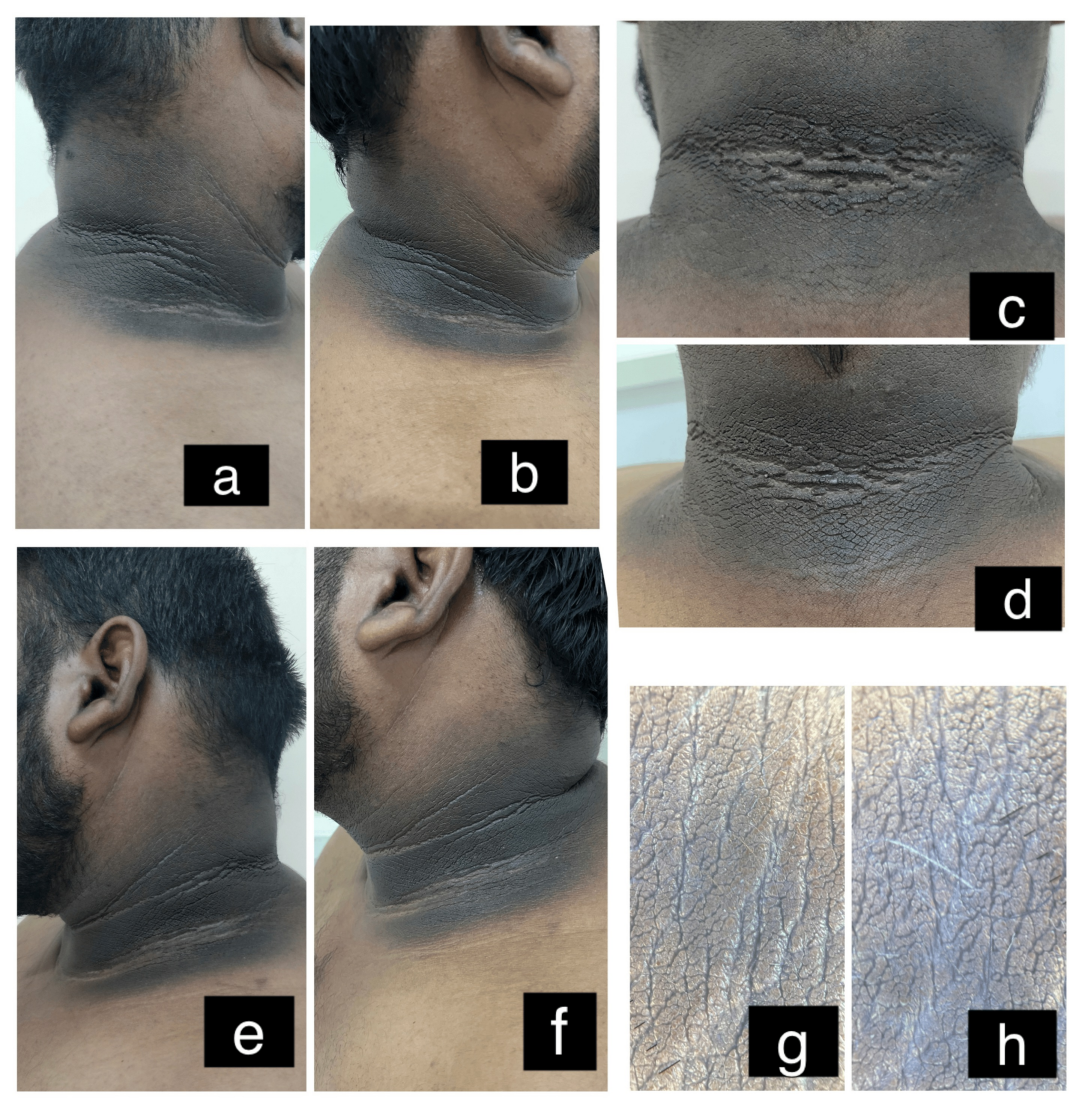
Before and after retinoic acid peel treatment a: Right side of the neck before treatment; b: Right side of the neck after treatment; c: Nape of the neck before treatment; d: Nape of the neck after treatment; e: Left side of the neck before treatment; f: Left side of the neck after treatment; g: Dermoscopic picture of the neck before treatment; h: Dermoscopic picture of the neck after treatment

On comparing the values, there was no significant difference (p=0.58) in the score between the groups before therapy. However, there was a significant difference (p<0.0001) noted in the score between the groups after therapy. Group A (Figure 3) showed superior results with a better outcome than group B (Figure 4).

In group A (fractional CO2 Laser), there was a significant (p=0.0001) fall in the ANASI score before and after therapy. Also, in group B (retinoic acid peel), there was a significant (p=0.0001) fall in the ANASI score before and after therapy. However, the fall in ANASI score that denoted the treatment response was greater in group A compared to group B, signifying superior results of fractional CO2 laser compared to retinoid acid peel.

Dermoscopic assessment

The improvement of dermoscopic signs such as cristae cutis, sulci cutis, and brown to dark brown dots were 16/19 (84%), 17/19 (89%) and 14/19 (73%) in the laser group and 4/19 (21%), 5 /19 (26%) and 3/19 (15%) in the peel group, respectively. None of the patients had milia-like cysts. There was a significant difference noted in the improvement of cristae cutis (p<0.001), sulci cutis (p<0.001), and brown to dark brown dots (p<0.001) between the groups, with the laser group showing a higher percentage improvement in results (Figures 3-4).

Adverse effects

In the laser group, three patients developed minimal side effects such as erythema, which is a common and expected reaction following laser skin treatment as a part of the normal healing process. The erythema resolved within a day with the application of topical corticosteroid (fluticasone propionate ointment 0.005%). One patient developed post-inflammatory hyperpigmentation, which was attributed to non-compliance with the recommended post-procedure care. In contrast, 10 patients in the peel group experienced intense pruritus, which subsequently led to post-inflammatory hyperpigmentation.

Assessment by the blinded dermatologists

**Figure 5:**
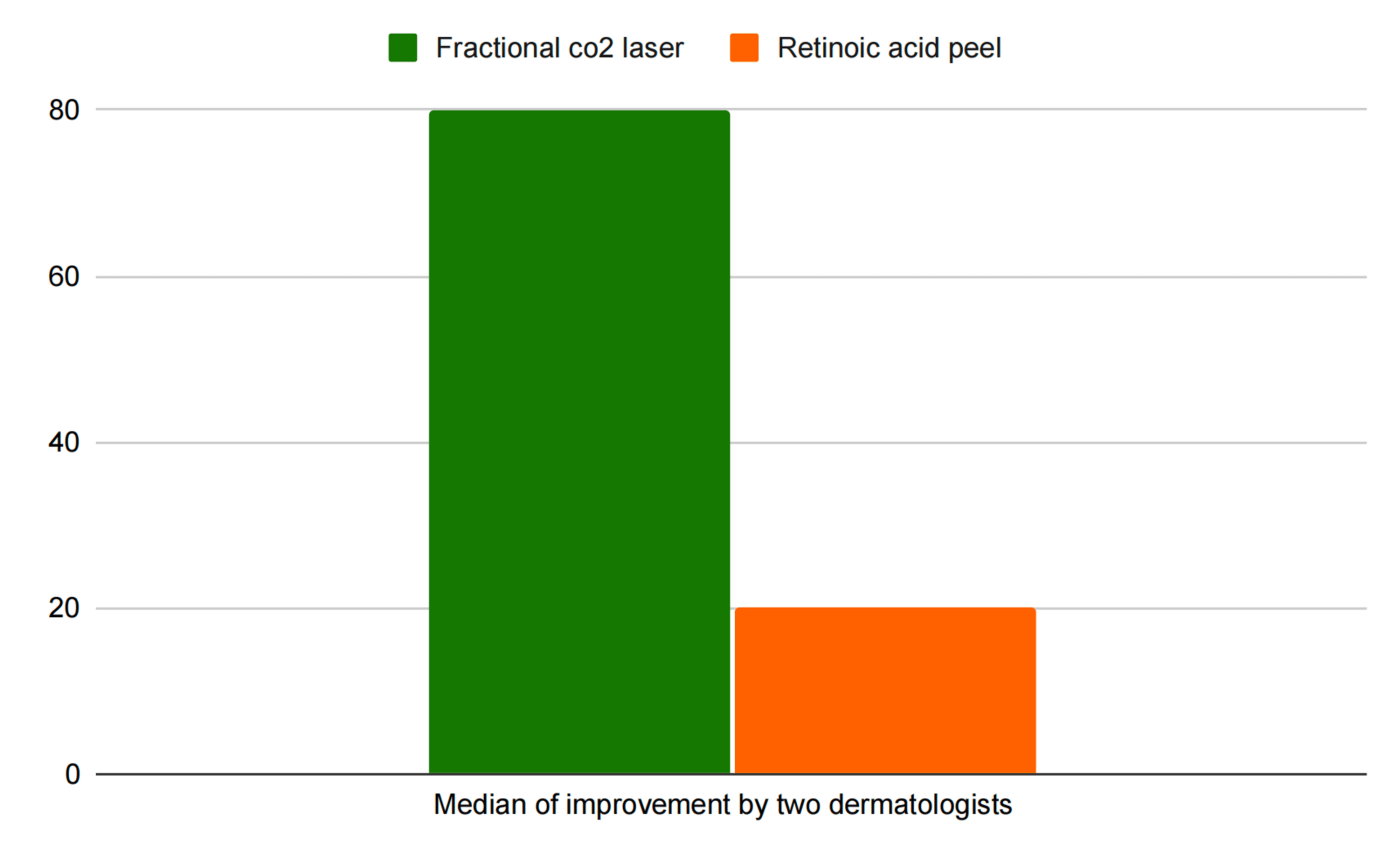
Median percentage of improvement of AN after the two forms of therapy as assessed by two blinded dermatologists AN: Acanthosis nigricans, CO2: Carbon dioxide

As described, two blinded dermatologists compared the clinical photographs taken before and after the therapy. They determined the median (IQR) improvement in group A (fractional CO2 laser) was 80% (IQR 75% to 85%) as compared to group B (retinoic acid peel), which had a median improvement of 20% (IQR 17.5% to 30%) as shown in Figure 5. This shows there is a significant difference (p<0.001) in median improvement between both the groups and fractional CO2 laser gives better results in treating AN of the neck than retinoic acid peel. 

## Discussion

Acanthosis nigricans is a cutaneous sign of an underlying disorder or disease. It is commonly seen in adults, but any age group can be involved. According to Curth’s classification, AN can be malignant, benign, inherited, pseudo-associated with obesity, and syndromic, whereas Schwartz described eight types of AN as benign, obesity-associated, syndromic, malignant, acral, unilateral, medication-induced, and mixed-type when two varieties are present [3]. The term pseudo-AN is now obsolete, as this condition differs only in degree from other forms. Acanthosis nigricans is a clinical diagnosis due to its classical appearance and doesn’t require any biopsy, but further evaluation for insulin resistance can be done [3]. Although weight reduction is helpful, only mild skin changes are appreciable, while skin thickening and pigmentation often persist.

The cutaneous lesion can be managed either topically or with systemic agents. Effective topical medications include keratolytics like topical tretinoin 0.05%, ammonium lactate 12% cream, podophyllin, urea, salicylic acid, and chemical peels like 15% trichloroacetic acid (TCA), retinoic acid peel, salicylic acid peel, glycolic peel [6]. Retinoic acid peel causes thinning and compression of the stratum corneum, cellular renovation, accelerating the cellular turnover of keratinocytes, reversal of epidermal cell atypia, dispersion of epidermal melanin pigment, neocollagenesis by stimulating collagen deposition in the dermis, increased glycosaminoglycan deposition, and new vascular formation [7]. Oral agents that have shown some benefit include etretinate, isotretinoin, and acitretin [6].

Dermabrasion and fractional ablative lasers like CO2 and erbium may also be used to reduce the bulk of the lesion, with some long-term remission. The fractional ablative laser improves textural irregularities and pigmented lesions by inducing a very narrow tissue column of thermal damage and ablation, aiming for dermal wound and neocollagenesis, which is the principal cause of improvement seen post-fractional laser resurfacing. The surrounding skin acts as a reservoir for healing, allowing rapid skin repair [8]. The fractional CO2 laser has emerged as a potential treatment in AN because of its ability to cause superficial skin surface ablation with trans-epidermal melanin elimination [9].

In our study, we compared fractional CO2 laser and retinoic acid peel and found that fractional CO2 laser gave impressive results with better outcomes as evidenced by the reduction in median (IQR) ANASI score from 20 (IQR 15-20) to 4 (IQR 4-8) which was statistically significant with a p-value of 0.0001. The fractional CO2 laser had a higher fall in ANASI score and a higher percentage of dermoscopic features improvement as compared to the retinoic acid peel. Besides improving the pigmentation and thickness, it also improved the skin texture. Adverse effects like post-inflammatory hyperpigmentation were minimal in the laser group compared to the peel group. The parameters of the fractional CO2 laser (Derma India, Chennai, TN, IND) used were power 10 W, dwell time 300 μs, spacing 350 µm, two stacking, and one pass.

Several trials have demonstrated favorable outcomes with the use of fractional CO2 laser in treating AN. In a study by Abu et al., the laser was found to be more efficacious than retinoic acid peel both clinically and dermoscopically, with a reduction in median ANASI score from 15 to 3 [4]. In a comparative study by Leerapongnan et al., the fractional 1550 nm erbium fiber laser was found to be significantly more effective than topical tretinoin cream in improving roughness and skin colour of AN lesions. All patients tolerated the laser treatment well, with no reported side effects. The reduction in roughness was 24.65% on the laser-treated group compared to 22.94% on the tretinoin-treated group, and this difference was statistically significant (p=0.004) [10]. Fatma et al. found that fractional CO2 laser therapy is a promising and effective treatment option for pseudo-AN, demonstrating higher clearance rates and fewer adverse effects compared to the TCA chemical peel. A highly statistically significant difference was observed between the clinical responses of the two treatments (p<0.01) [11].

Similar findings were reported by Fouda et al., where fractional CO2 laser therapy was compared with 20% TCA peel. The fractional CO2 laser group demonstrated a higher percentage of improvement, with a statistically significant difference in disease response between the two groups (p=0.001) [12]. In another study on unilateral AN by Campos et al., fractional CO2 laser yielded satisfactory results [13]. In a study by Zaki et al., fractional CO2 laser was compared with glycolic acid peel. Although the latter demonstrated the advantage of earlier clinical improvement, the fractional CO2 laser was associated with skin tightening and a more sustained, long-term improvement in skin texture [8]. Additionally, other laser modalities such as Q-switched neodymium-doped yttrium aluminum garnet (Nd:YAG) and potassium-titanyl-phosphate (KTP) lasers have also been found to be effective and safe treatment options for AN [14].

Conversely, few studies have highlighted the efficacy of topical tretinoin in the management of AN. In a study by Treesirichod et al., the daily application of 0.05% tretinoin cream led to notable improvement in hyperkeratosis, although some patients experienced skin irritation as a side effect [15]. Similarly, Srinivas et al. found that topical 0.025% tretinoin cream was more effective in reducing the severity of AN compared to retinoic acid peel treatments (administered at 10% followed by 4%), suggesting that tretinoin remains a promising topical therapeutic option for AN [16].

Similarly, Milosheska et al. highlighted the differences in the side effect profiles between topical tretinoin 0.025% cream and 4% retinoic acid peel, noting that while the peel was associated with fewer side effects, the topical tretinoin cream yielded higher patient satisfaction [17]. In contrast, in our study, we used a 4% retinoic acid peel for only four sessions at three-week intervals, and none of the patients experienced any cutaneous irritation, indicating good tolerability with this treatment protocol.

The main strength of our study lies in its randomized controlled design, along with the use of both clinical and dermoscopic assessments of AN, which enhances the reliability of the findings. However, the study has some limitations, including a small sample size, being restricted to a single locality, and a short duration of follow-up. Conducting a study with a larger sample size and long-term follow-up would provide a more comprehensive understanding of the differences between the two treatment modalities in AN.

## Conclusions

This study concludes that fractional CO2 laser is more efficacious than retinoic acid peel in the treatment of AN, particularly in improving lesion appearance, skin texture, pigmentation and skin tightening, as demonstrated through both clinical photography and dermoscopy. The outcomes were also found to be sustainable on follow-up. Furthermore, fewer laser sessions were required to achieve effective results and side effects were minimal, likely due to the use of low-power laser parameters that still delivered the desired therapeutic effect. Based on these findings, we recommend fractional CO2 laser as a superior and safe treatment modality for AN.
